# Relationship between Molarity and Color in the Crystal (‘Dramada’) Produced by *Scytalidium cuboideum*, in Two Solvents

**DOI:** 10.3390/molecules23102581

**Published:** 2018-10-09

**Authors:** Sarath M. Vega Gutierrez, R. C. Van Court, Derek W. Stone, Matthew J. Konkler, Emily N. Groth, Seri C. Robinson

**Affiliations:** 1 Department of Wood Science & Engineering, Oregon State University, Corvallis, OR 97331, USA; ray.vancourt@oregonstate.edu (R.C.V.C.); konklerm@oregonstate.edu (M.J.K.); seri.robinson@oregonstate.edu (S.C.R.); 2 Bioengineering, Oregon State University, Corvallis, OR 97331, USA; stonede@oregonstate.edu; 3 Materials Science and Engineering, Washington University in St. Louis, St. Louis, MO 63130, USA; egroth@wustl.edu

**Keywords:** *Scytalidium cuboideum*, Dramada, saturation, molarity, color difference, fungal pigment

## Abstract

Pigments from wood-decay fungi (specifically spalting fungi) have a long history of use in wood art, and have become relevant in modern science due to their longevity and colorfastness. They are presently under investigation as colorants for wood, bamboo, oils, paints and textiles. Major hurdles to their commercialization have been color repeatability (in that the same strain of the same species of fungus may produce different colors over time), and the binding of the pigments to glass storage containers. This is persistent as they do not naturally exist in a loose form. Due to these issues, the ‘standard’ color for each was historically determined not by the amount of pigment, but by the color in a solution of dichloromethane (DCM), using the CIE L*a*b colorspace. This method of standardization severely limited the use of these pigments in industrial applications, as without a dry form, standard methodologies for repeatable color processing into other materials could not be easily implemented. Recent studies have developed a method to crystalize the red pigment from *Scytalidium cuboideum* (Sacc. & Ellis) Sigler & Kang, producing a highly pure (99%) solid crystal named ‘Dramada’. Herein a method is detailed to compare the molarity of this crystallized pigment to variations in the color, to determine a color saturation curve (by weight) for the pigment from *S. cuboideum* in DCM and acetone. The molarities for this experiment ranged from 0.024 mM to 19 mM. Each molarity was color read and assigned a CIEL*a*b* value. The results showed that there was a correlation between the molarity and color difference, with the maximum red color occurring between 0.73 mM and 7.3 mM in DCM and between 0.97 mM to 0.73 mM in acetone. Extremely low molarities of pigment produced strong coloration in the solvent, and changes in molarity significantly affected the color of the solution. Having a saturation and color curve for the crystal ‘Dramada’ from *S. cuboideum* will allow for the reliable production of distinct colors from a known quantity (by weight) of pigment, erasing the final hurdle towards commercial development of the crystallized pigment from *S. cuboideum* as an industrial dyestuff.

## 1. Introduction

Pigments derived from fungi have been used as dyestuff since ancient times [1,2,3,4,5]. With uses ranging from coloring textiles [4] to food [6], fungal pigments are both versatile and abundant in nature [5]. Of these pigments, the ones produced by spalting fungi [7,8,9] (fungi that color wood internally) are of special interest because of their unique colorfastness and the corresponding industrial applications in textiles, wood dyes, and finishes.

Spalting fungi are classified into three categories based on their effects on wood [9]. The first is bleaching, caused by lignin degrading white rot fungi which are mostly Basidiomycetes [10,11]. The second category is characterized by zone line production, which are pigmented thin winding lines in wood [12,13]. These are produced in response to somatic incompatibility [14,15,16,17] or dehydration [12], and are mostly composed of melanin [18,19,20], giving them their characteristic black coloration. Competing Basidiomycetes are the most common zone line producers [8,21], but Ascomycetes can form them as well. For example, fungi from the genus *Xylaria* are some of the most prolific zone-line producing fungi [22].

Pigmenting fungi make up the third and final category [9]. These fungi are soft-rot Ascomycetes [23] that produce pigments as secondary metabolites [24]. The primary pigments of interest in this category are the yellow pigment (produced by *Scytalidium ganodermophthorum* Kang, Sigler, Lee & Yun) [25,26], a blue-green pigment (produced by the genus *Chlorociboria* spp. Seaver ex C.S. Ramamurthi, korf & L.R. Batra) [27,28,29] and a red pigment (produced by *Scytalidium cuboideum* (Sacc. & Ellis) Sigler & Kang [30,31]. These fungal pigments have naphthoquinone-like [3,32] chemical structures and have shown great potential in use for industrial applications. For example, xylindein [27,33,34,35,36,37] (blue-green coloration), produced by *Chlorociboria* spp., is currently being researched for solar energy applications [38]. This pigment has also shown outstanding properties in use as wood [39,40] and textile [41] dyes. Another pigment that has been characterized is draconin-red [42], the sole known pigment produced by the fungus *Scytalidium cuboideum*. This pigment forms crystals [43] and has shown strong color stability when used in paints [44,45], textiles [42,46] and in the wood industry [39,40] as dyestuffs. The yellow pigment of *Scytalidium ganodermophthorum* is still undescribed and under research.

One of the most unique aspects of the aforementioned pigments is their solubility. Spalting pigments are found in the area around hyphae in fungal colonies, which would suggest some form of diffusion is taking place. However, they have been found to have very limited solubility overall. A previous study [47] found that non-polar solvents such as dichloromethane (DCM), tetrahydrofuran (THF), chloroform, and acetone were capable of extracting the pigments from wood, although all but DCM altered the color of the pigment [39,40,47]. This has made DCM the preferred pigment carrier for further laboratory experimentation, where toxicity issues associated with it can be abated. The pigments use as dyestuff for wood has been studied with different DCM application methods, such as dripping [40] and pressure treatment [39]. Both tests showed promising results for the external coloration of wood. Similar studies were performed on textiles, where the pigments showed an outstanding capacity for polyester coloration, [41] exceptional colorfastness, and resistance to UV-light exposure [42,46,48]. Bamboo was also tested [49] yielding similar results as those for wood.

While the demonstrated uses of the pigments suggest they could be used as an environmentally friendly alternative to traditional coloring practices, unfortunately the inherent toxicity of DCM limits their applications especially within large-scale industries [50]. Further experimentation has determined that the pigments could be carried by oils [45], allowing research into the application as dyes in oil and acrylic paints [44] and as oil-based textile dyes [51]. Acetone has also been proven to be capable of carrying the red pigment produced by *Scytalidium cuboideum,* which has been demonstrated via the use of acetone in extraction of the pigment from wood chip plates [47], as it is also the solvent used in the production of the pure crystals [43]. As acetone is a common solvent used in a variety of industrial applications [52,53,54], it may represent the best alternative to DCM, but only if it can perform comparably.

The use of color values as a measure of pigment quantity has been standard for all the aforementioned publications related to these pigments. A color standard (CIEL*a*b* color space) has been used as an indirect measure of pigment quantity instead of dry weight. The standard was based upon the average color produced by the extraction of one fungal plate culture extracted in 50 mL of DCM [40]. These extractions produced variable color, even between fungi from the same strain, so the color standard using CIEL*a*b values was adopted to achieve evenness in pigmentation [40].

Use of the ‘standard’ solution was developed to address two main issues with spalting fungal pigments. The first was the inherent color variability between not only strains, but also plates. A given amount of pigmented media from one plate of fungus could have one color, and the same amount from another plate of that same strain have a different color, due to different environmental stressors and micro changes in growing conditions. As such, the amount of pigmented media used to make pigment solutions was not a reliable method to determine color. This matter could have been resolved by the collection and usage of solid pure pigment after extraction. There was a second issue, however. It has been observed during the evaporation of the solvent that the pigments bind to their glass containers and cannot be collected in a solid form without further treatments. This particular solubility issue has prevented the obtaining of pure, solid forms of pigments from any of the aforementioned species [40]. However, recently a method to crystallize the pigment from *S. cuboideum* was developed [43,55], allowing for the collection of an almost pure (99%) form of the crystal [43]. The fact that there is now a solid form allows for the production of solutions with known concentration, the color of which can then be compared to the standard used in past publications in order to identify actual quantities required for tested applications.

The ability to obtain a known concentration of pigment is of critical importance for measures of pigment toxicity, as is knowing the amount of pigment needed to impart color in order to identify reasonable testing ranges [56]. Because of this, previous experiments into spalting pigment toxicity have had limited utility. This was because they used the differences in chromatic values (CIE L*a*b* − ΔE) between pure solvent and pigmented solvent as an indirect measure of pigment quantity, which was never related to actual pigment concentration [56]. In order to perform more effective tests on toxicity, having a solid form of the pigment allows for the understanding of the relationship between pigment quantity and produced color.

To enable the potential industrial use of pigments produced by spalting fungi, this paper deals with the relationship between the changing molarity of the pigment produced by *Scytalidium cuboideum* and the color difference values when carried in DCM and acetone. The information generated will allow for the development of formulas that can relate pigment concentration to CIEL*a*b* values. It will also give a definitive weight-to-color (M to CIEL*a*b) relationship that will lend greater utility to previous studies that deal with this pigment, such as allowing for critical economic feasibility analyses. This experiment also sets forth methods that can be used to determine the concentration and relationship to color difference for the extracted pigments of other spalting fungi pigments such as *Chlorociboria* spp. and *S. ganodermophthorum* in the future.

## 2. Results

The molecular purity of the pigment was confirmed with the use of NMR, as it was the method that was previously applied for determining the chemical composition of the fungal crystal [43]. With it, a NOESY identification spectra was obtained, signaling the same peaks as the previously identified compound. No peaks indicating impurities, such as fatty acids, were present on the spectra. This allowed us to determine the purity of the pigment at 99%. A similar result was observed with the use of GC-MS, which showed little interference within the peaks. The compound purity was taken into account for the molarity calculations. To view the NMR spectra, please go to Supplementary Materials Figure S1, and for the GC-MS spectra. Please go to Supplementary Materials Figures S2 and S3.

**Figure 1:**
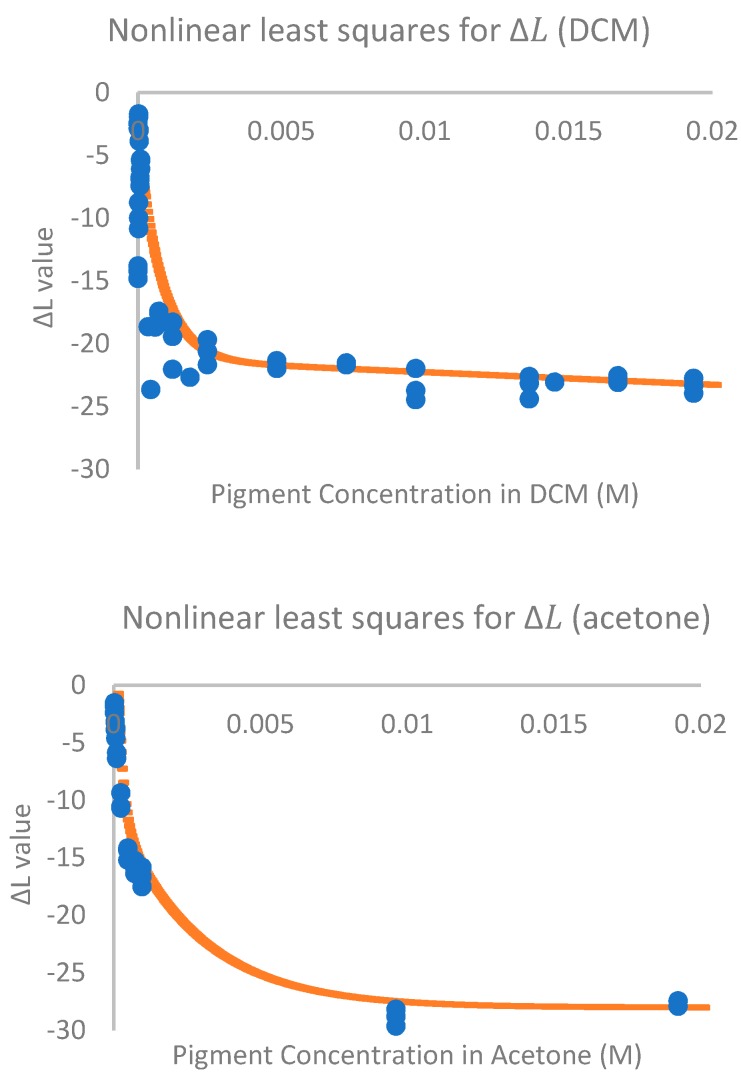
Molarity vs. ΔL Color Value for the crystal ‘Dramada’ from *Scytalidium cuboideum.* The upper image corresponds to DCM and the bottom one to acetone.

The shape of the curves produced by varying concentrations of pigment and ΔL values was found to be similar for both DCM and acetone solutions (Figure 1), but due to their different solubility relationships with the pigment they have been analyzed separately [47]. ΔL values were selected for non-linear regression modeling on the basis of their one-to-one relationship with molarity that other color read values did not possess. For DCM solutions the line of best fit was determined to be y = −106.3^(0.924 × x)^ + 15.08^(−1218 × x)^ + 88, with an R^2^ value of 0.86. For acetone the line of best fit was calculated as y = 11.97^(−5392 × x)^ + 16.64^(−360.7 × x)^ − 28 with an adjusted R^2^ value of 0.99. These equations fit well with observed saturation points. At high molarities the solution was fully saturated and crystals formed on the glass after inversion, though they went back into solution and formed again on further inversions. This was no longer observed in solutions of 19 mM for DCM and 9.7 mM for acetone, suggesting dilution had gone past saturation points, a difference that is reflected in the models. These developed equations are reflective of observed color changes, and will allow the quantification of the amount of pigment in solution from an extracted culture in DCM or acetone using ΔL values.

**Figure 2:**
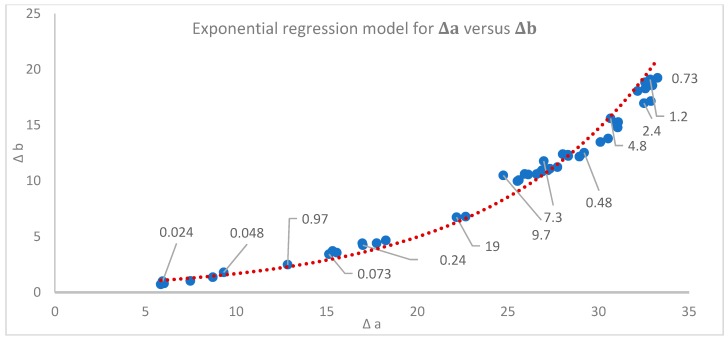
Exponential regression model for the color change relation between Δa and Δb color values for the crystal ‘Dramada’ in DCM. Labeled values are the corresponding pigment concentrations (mM).

Interestingly, increasing concentration was also associated with shifts in hue of the solution. The relationship between Δa and Δb was modeled with an exponential regression, giving an equation of Δb = 0.5699e^0.1083^^Δ^^a^ (R^2^ = 0.98032) (Figure 2). In the CIE L*a*b color space, increasing Δa values are associated with increased red coloration, and increasing Δb values are associated with greater yellow coloration.

**Figure 3:**
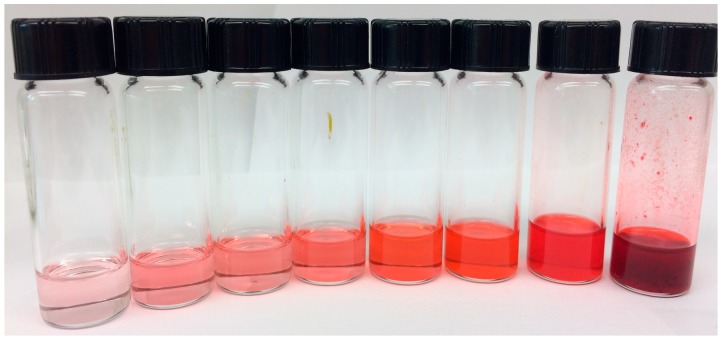
Varying Concentrations of the crystal ‘Dramada’ from *Scytalidium cuboideum* in DCM. Solution concentrations range from 0.024 mM to 19 mM of the pigment (**left** to **right**).

The color shift was not only quantified, but it was possible to visually assess the color change of the different concentrations (Figure 3).

In addition to charting change in color, the points at which there was a significant color difference due to molarity were identified using ΔE values. An ANOVA showed a significant difference between tested concentrations in DCM (*p* < 0.0001) with a Tukey HSD test identifying which color values were significantly different from each other. Molarities between 0.73 mM and 7.3 mM were found to be equivalent and had the highest ΔE values, representing the greatest difference from the solvent, and can be considered the most intensely pigmented (Figure 2). More detail on the groups can be seen in Table 1.

The relationship between the highest Tukey mean ΔE group L*a*b* values (Table 2) and the color values used to create standard solutions for the pigment from *S. cuboideum* is also informative.

Acetone also exhibited a significant (*p* < 0.0001) relationship between molarity and ΔE values. When categorized with the Tukey HSD test, the concentrations belonging to 0.97 mM and 0.73 mM showed the greatest mean ΔE values (Table 3).

**Figure 4:**
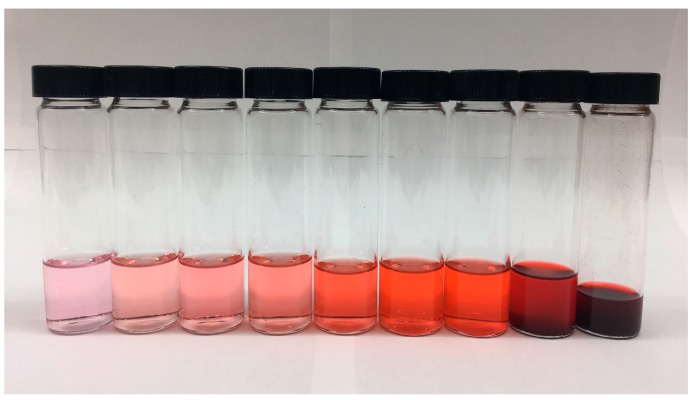
Varying concentrations of the crystal ‘Dramada’ from *Scytalidium cuboideum* in acetone. Solution concentrations range from 0.024 mM to 19 mM (**left** to **right**).

The different concentrations in acetone showed a visible color difference (Figure 4), as previously observed with the DCM concentrations.

## 3. Discussion

The relationship between Δa and Δb in DCM (Figure 2) showed that with an increase of the red color, there was an exponential increase in the yellow coloration as well. Colors moving towards yellow as they become lighter is a trend that has been observed by color modeling, [57] which likely contributed to this effect. When these values are compared to ΔL values, trends emerge. The lowest Δa values were associated with the most diluted solution with lowest ΔL (least darkening of color), with Δa values increasing with a concentration until a Δa value of 23. After this point Δa was associated with the highest ΔL and concentration values. Interestingly, the highest values on the Δa vs. Δb chart were associated with the middle ΔL values, which correspond with molarities of 7.3–2.4 mM. When compared visually (Figure 3), the solutions at these concentrations had a noticeable shift towards an orange coloration. The change in the hue associated with changing concentration in addition to the lightening of the color is an interesting effect, and could be used for the production of a more diverse range of colors.

It is also important to note that even for the lowest molarities the mean ΔE values show differences with values greater than ΔE=2.3. This ΔE value has been previously reported as the point where the human eye can actually differentiate the chromatic difference between colors [39,58]. The ΔE values therefore confirm visual observations (Figure 3) that the concentrations in DCM ranging from 0.024 mM to 0.48 mM can be perceived as different colors, while the molarities from 0.73 mM to 4.8 mM and those between 7.3 mM to 19 mM have little difference. The same holds for acetone, where concentrations between 0.024 mM and 0.73 mM can be perceived as different colors though between 0.73 mM and 0.97 mM and those between 9.7 mM and 19 mM would not be. In addition, the fact that there is a mean ΔE of 6.26 (for DCM) and 4.39 (acetone) for the lowest molarity tested (0.024 mM), indicates that there is a visible color variation between solvents. This result is important for future industrial applications, as it indicates that the required amount of crystallized pigment from *S. cuboideum* to generate a visible color change depends on the solvent. A statistical comparison between the color difference of the pigment carried in DCM and acetone was not done, but previous experimentation performed by Robinson et al. (2014) [47] showed that based on ΔC ($∆C_{ab}^{*}=C_{1}^{*}-C_{2}^{*},\;where\;C_{x}^{*}=\sqrt{a_{x}^{{*}^{2}}+b_{x}^{{*}^{2}}}$) values, the resolubilization of dry pigment (bound to glass) performed moderately well with acetone (not all the pigment was resolubilized) compared to the complete pigment resolubilization in DCM. It therefore follows that the solubility of the crystalized pigment was lower in acetone than in DCM, which could affect the final color difference.

In previous publications, the color value for standard pigment solution was set at $L^{*}=82.32\;\left(\pm 2\right),\;a^{*}=26.84\;\left(\pm 2\right),\;\mathrm{and}\;b^{*}=13.19\;\left(\pm 2\right)$ for direct extractions with DCM from culture plates [39,40,42,49]. These values are similar to those that gave the largest ΔE values, suggesting that the standard concentration that has been used is also the most color-saturated solution. Based on the proposed model built on ΔL, is possible to theorize that the previously used standard concentration fell within the range of 0.73 mM to 2.4 mM.

For both solvents, the characterization of the relationship between color and concentration as measured by molarity for the pigment of *S. cuboideum* is highly valuable. The relationship between mol·kg^−1^ or mol·L^−1^ is required for different formulations across industry [59,60], and the ability to obtain those values directly from extracted pigment in solution opens the door for industrial use. The relationships modeled between molarity and ΔL, Δa and Δb values also have the future potential to model the variety of hues that could be obtained by the pigment. Modeling has been used elsewhere to obtain color palettes for a variety of colors [57], utilizing contrast and saturation elements that could be introduced in future work to obtain detailed coloration predictions.

In addition, previous research conducted with the pigment of *S. cuboideum* can now be understood in terms of the actual quantities of pigment used, instead of depending on a chromatic standard. This can give a more accurate overview of the concentration of the pigments used for textiles [41,42,46,51], wood [39,40], bamboo [49], paints [44], oils [45] and for toxicology analysis [56]. In addition to allowing more specific research in the future, it will also aid in the development of economically feasible applications, as it has been shown that the quantities of pigment required for coloration can be lower than 0.024 mM (0.0605 mg/L).

## 4. Materials and Methods

### 4.1. Pigment Production

The fungus *Scytalidium cuboideum* UAMH 11517 isolated from treated *Quercus* sp. in Memphis, TN, USA, was cultivated on 2% malt agar (VWR) amended plates with white-rotted maple wood chips, following the protocol set by Robinson et al. (2012) [29]. The fungi were grown for six weeks to obtain a high yield of pigment.

After the six weeks, the plates containing *S. cuboideum* were opened and dried under room temperature conditions (20 °C) in a fume hood for 24 h. The dry wood chips containing the pigment were then ground for 15 s, utilizing an Oster (model 6811) blender. After this process, 200 g of chips were placed in a 2 L Erlenmeyer flask (VWR) with 500 mL of 99.9% HPLC acetone (VWR) with a stir bar (VWR). The flask containing the mix was then placed on a stir plate (VWR) at 400 rpm for 15 min.

After the stirring time was completed, the mix of acetone and wood chips was filtered using a Pyrex filtering funnel equipped with a 415 filter paper (VWR) in a 1 L VWR flat-bottom boiling flask. The solution was then reduced to 50 mL using a Büchi rotovap, model 461, in a 40 °C DI water bath.

### 4.2. Crystallization

The crystallization process was done following the methods stated by Vega et al. (2018) [43]. The reduced solution was placed in a 500 mL Erlenmeyer flask with 100 mL of liquid nitrogen. The solution was then stirred slowly by hand, until a precipitate was formed after which the solution was filtrated into a new flask utilizing a Pyrex filtering funnel equipped with a 415 filter paper. The collected precipitate, which corresponds to the crystal form of the pigment (‘Dramada’), was left to dry on the filter paper in a fume hood at room temperature (20 °C) to allow the remaining acetone to evaporate.

Once the filter paper was completely dry, the crystals were recovered using curved dissecting forceps (VWR) by peeling them from the surface of the filter paper, and placed into a 30 mL glass sample vial (Ace Glass Incorporated, Vineland, NJ, USA).

The chemical identity and the purity of the compound were confirmed using nuclear magnetic resonance (NMR) and gas chromatography–mass spectrometry (GC-MS). For NMR, the data was collected with the use of a Bruker AVIII 700 (Bruker, Portland, OR, USA), running Topspin 3.5PL7. The probe head employed was a DCH cryo-probe, Dual Nucleus C13 and H1 with H2 lock and Z gradient.

For GC-MS, the crystals were diluted in HPLC grade DCM at a concentration of 100 mg/mL. Then, they were placed in a Shimadzu QP2010S GC-MS (Kyoto, Japan) operated in scan mode, mz range 45–900, with a splitless injection. One µL of sample was injected and analyzed with the following GC conditions: The temperature program was 80 °C for 2 min, then ramped 10 °C/min until 190 °C was reached, then ramped 15 °C /min until 200 °C and held for 5 min, lastly it was ramped to 300 °C at 10 °C/min and held for 14 min. (total run time 40 min.), ion source temperature: 260 °C, interface temperature: 250 °C, injection temperature 250 °C. The sample was analyzed on an RXI-5ms column (0.25 mm inner diameter by 30 mm long) at a flow rate of 1.2 mL/min. The extractive present in the resulting chromatogram was identified using the National Institute of Science and Technology (NIST) Mass Spectral Library #107 software (Gaithersburg, MD, USA).

### 4.3. Molarity Calculation and Resolubilization

The chemical composition of the crystallized pigment of *S. cuboideum* (‘Dramada’) is 3,6-di-methoxy (di-hydroxyl-napthoquinone) (C_12_O_6_H_10_) with a total molecular weight of 250.20 g/mol [43]. Solutions of different molarity were prepared in both 10 mL of dichloromethane (DCM) and 10 mL of HPLC grade acetone (Table 4). To prepare the solutions, crystals were weighted into a 30 mL glass sample vial using a Veritas precision balance (H&C Weighing Systems model M124A) after which 10 mL of DCM or acetone were placed in the vials. Vials were capped and then inverted 30 times, or until crystals were completely solubilized. The most concentrated solution (19 mM) was made up first, then diluted to produce solutions of lower molarity with the aim of reaching L*a*b values used in previous papers [39,40,42,44,46,49,56,61]. An overall volume of 10 mL was maintained, with dilutions continuing until a curve was reached. Additional readings were taken for DCM at concentrations of 17 mM, 15 mM, and 12 mM during the initial phase of the experiment when the curve was still being located, though these readings were not done for acetone.

Additional samples were made up in only DCM solution to investigate possible color changes at concentrations higher than 19 mM, starting with a 100 mM solution in 3 mL of DCM. After data collection, an additional one milliliter of DCM was added to the solution until a mixture within range of the starting 19 mM was obtained. Tested concentrations included 100 mM, 77 mM, 52 mM, 44 mM, 39 mM, 34 mM, and 26 mM.

### 4.4. Color Difference

Prepared solutions in each solvent (DCM and acetone) were individually color read in a Konica Minolta Chromameter CR-5 using a three milliliter VWR glass cuvette. Each solution had three repetitions, excluding those tested at high concentrations. These saturated solutions were done with one repetition as these were intended only to ensure the saturation point had been reached, and were not used in statistical analyses. The color difference (ΔE) was obtained by comparing each solution against the corresponding solvent (DCM or acetone) control, and was calculated utilizing the CIE76 formula. This formula is more suitable for measurements in liquids than CIE00, and has been used in previous studies [39,40,49,56]:

(1) $${\Delta E}_{76}^{*}=\sqrt{{\left(L_{2}-L_{1}\right)}^{2}+{\left(a_{2}-a_{1}\right)}^{2}+{\left(b_{2}-b_{1}\right)}^{2}}$$

### 4.5. Data Analysis

A one-way ANOVA followed by Tukey HSD test was performed on SAS version 9.4 for each test. For evaluating the data obtained, the independent variable was molarity, and the dependent variable was color difference (ΔE).

A nonlinear regression was performed with Matlab’s (Version 2017a) curve-fitting tool, based on the relationship between the ΔL values and concentration. ΔL values represent where the color value falls on a scale from white to black, and most accurately represented the change in concentration. The nonlinear least squares method was performed with a trust-region algorithm. All models converged before 400 iterations, save for the model for acetone, which converged by 1000 iterations. R^2^ values were calculated by Matlab using R^2^ = 1 − SSE/SST as a measure of practical fit of the model.

## 5. Conclusions

The relationship between concentration (M) and color values in the CIE L*a*b color space were successfully described for the pigment of *S. cuboideum*. It was also observed that extremely low molarity of pigment could produce strong coloration in both DCM and the less toxic acetone. In addition, different molarities produced variation in color not only in terms of lightness, but also in terms of hue. These characteristics further demonstrate the versatility of the pigment, as it can be used to produce a wide color palette and easily measured out to obtain reliable colors, and only small quantities are required to produce strong coloration, all of which suggest promising future industrial use.

## Figures and Tables

**Table 1:** Mean ∆E of the molarities when dissolved in DCM. Different letters classify the data as statistically different within each molarity. DF = 15.

Solvent	Molarity (mM)	Mean ∆E	Standard Deviation
DCM	0.024	6.26 (G)	0.06
	0.048	9.13 (G)	0.97
	0.073	19.03 (F)	0.54
	0.097	16.01 (F)	1.27
	0.24	23.92 (E)	2.82
	0.48	34.34 (D)	0.56
	0.73	41.87 (A)	0.56
	1.2	41.72 (A)	0.70
	2.4	40.97 (A)	1.71
	4.8	40.72 (AB)	0.30
	7.3	38.91 (ABC)	1.20
	9.7	37.14 (BCD)	2.29
	14	36.93 (DC)	1.07
	15	37.19 (BCD)	0.41
	17	36.21 (DC)	1.29
	19	36.81 (DC)	0.70

**Table 2:** Mean L*a*b* values for the Tukey HSD (A) group of *S. cuboideum* pigment in DCM.

Molarity (mM)	L*	a*	b*
0.73	82.14	32.72	18.99
1.2	81.13	32.40	18.23
2.4	80.26	31.85	16.51

**Table 3:** Mean ∆E of the molarities when dissolved in acetone. Different letters classify the data as statistically different within each molarity. DF = 9.

Solvent	Molarity (mM)	Mean ∆E	Standard Deviation
Acetone	0.024	4.39 (H)	0.18
	0.048	8.26 (G)	0.14
	0.073	11.35 (F)	0.79
	0.097	14.93 (E)	0.08
	0.24	26.21 (D)	1.78
	0.48	35.88 (B)	0.25
	0.73	38.20 (AB)	0.49
	0.97	39.36 (A)	1.38
	9.7	29.64 (C)	0.72
	19	28.32 (DC)	1.50

**Table 4:** Solution concentrations (mM) in both DCM and acetone.

Solution Concentration (mM)
19
9.7
0.73
0.48
0.24
0.097
0.073
0.048
0.024

## Supplementary Materials

The following are available online, Figure S1: NOESY (CDCl_3_) spectrum of ‘Dramada’, Figure S2: GC-MS data of ‘Dramada’, Figure S3: GC-MS data indicating the purity of the crystalline material used for the experiment.
