# Supplementary material for: Relationship between Molarity and Color in the Crystal (‘Dramada’) Produced by Scytalidium cuboideum, in Two Solvents
Source: Molecules. 2018 Oct 9;23(10):2581. doi: 10.3390/molecules23102581 (PMC6222885; doi:10.3390/molecules23102581)
Supplement: Supplementary file 1 [file molecules-23-02581-s001.pdf]

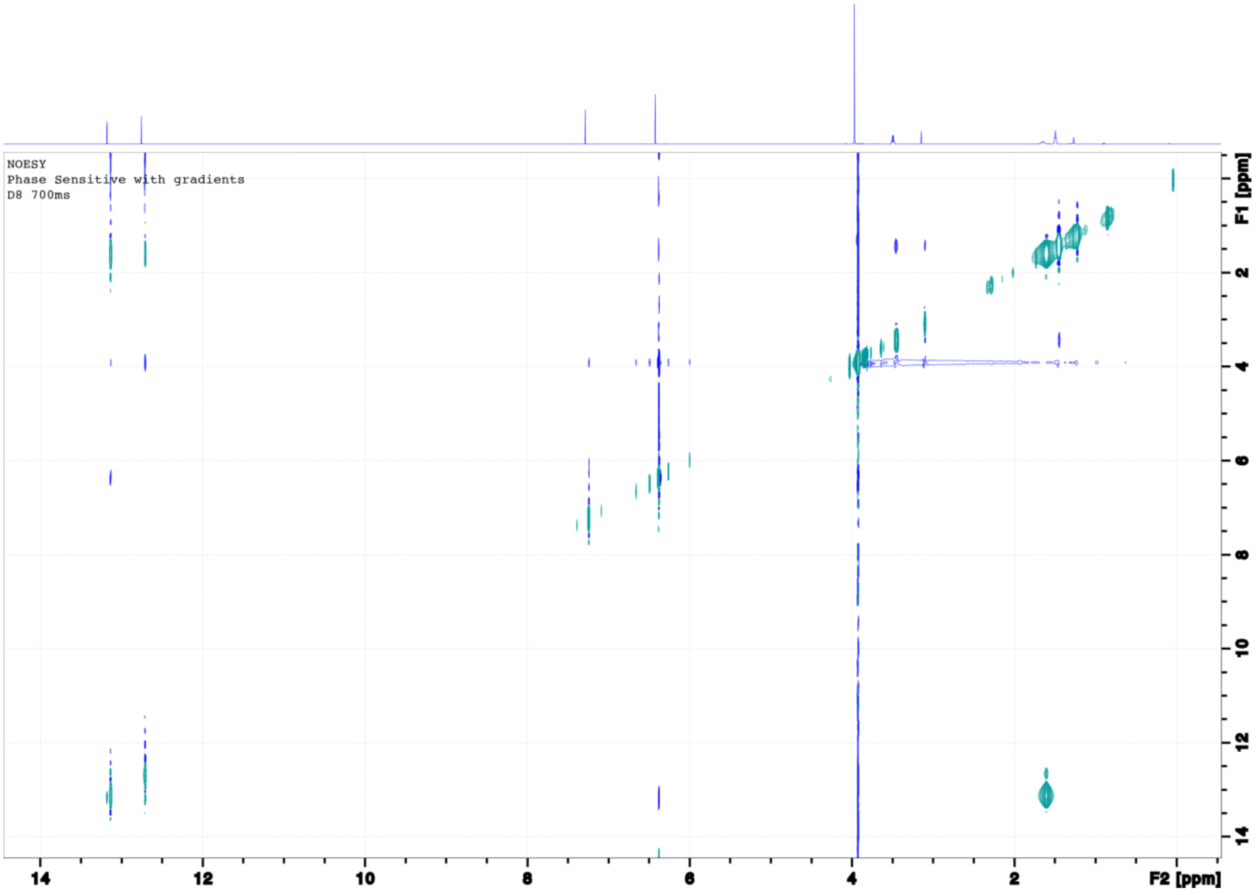

Figure S1. NOESY ($CDCl_3$) spectrum of 'Dramada'.

Supplementary Materials

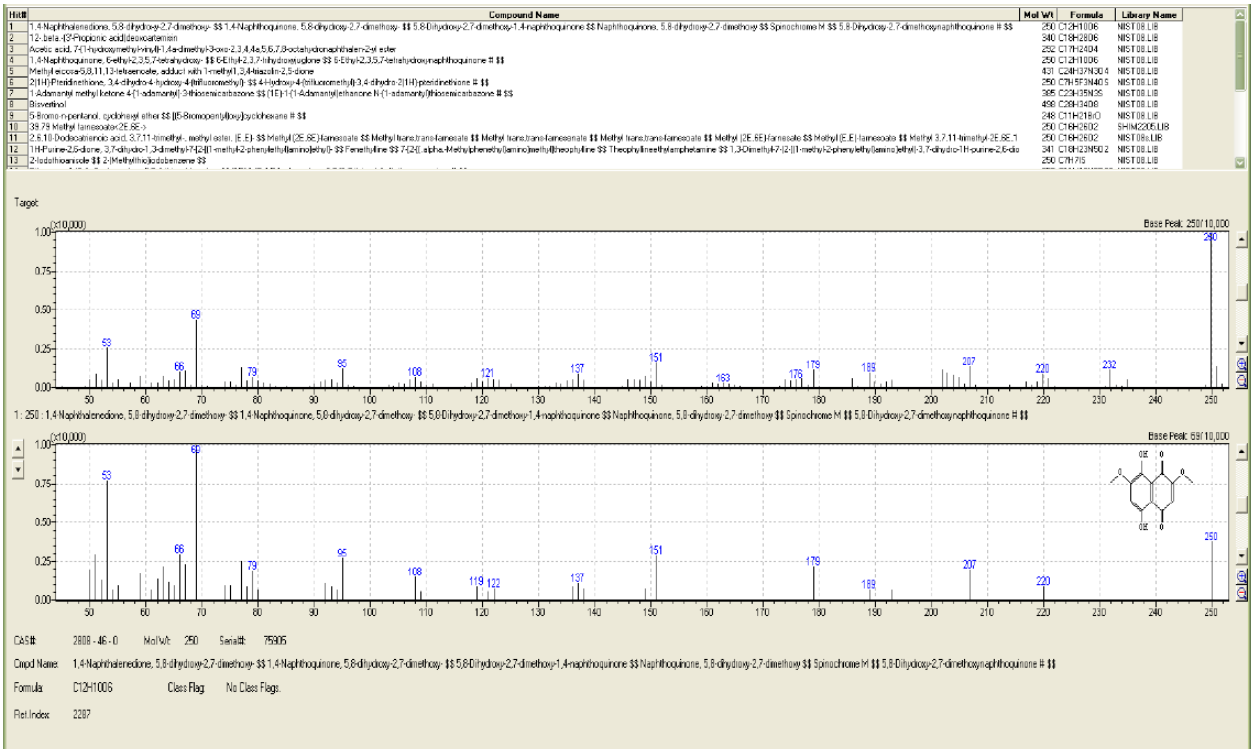

Figure S2. GC-MS data of ‘Dramada’.

Supplementary Materials

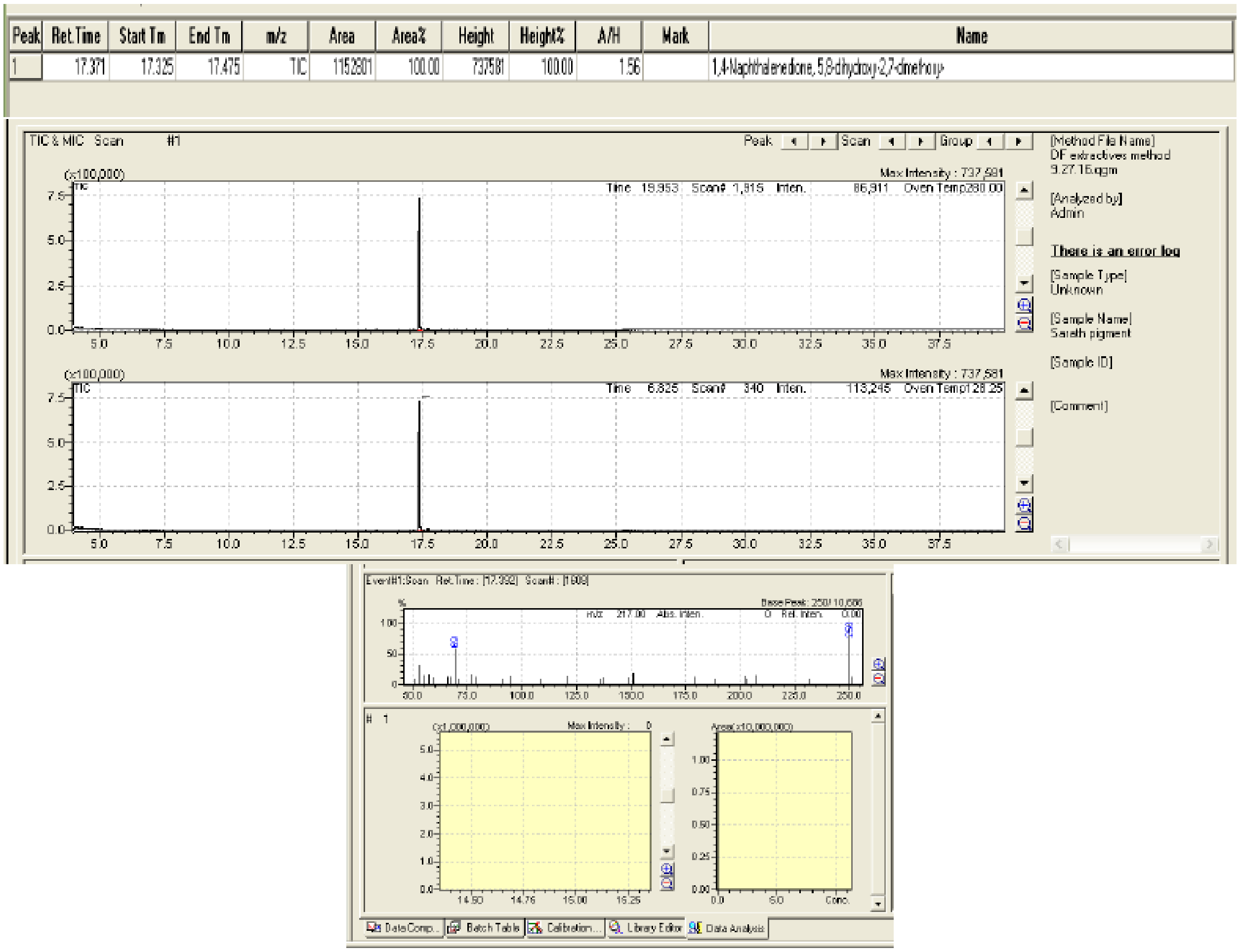

Peak	Ret.Time	Start Tm	End Tm	m/z	Area	Area%	Height	Height%	A/H	Mark	Name
1	17.371	17.325	17.475	TIC	1152801	100.00	737581	100.00	1.56		1,4-Naphthalenedione, 5,8-dihydroxy-2,7-dimethoxy-

Figure S3. GC-MS data indicating the purity of the material used for the experiment.
